# Targeting CCR2 with its antagonist suppresses viability, motility and invasion by downregulating MMP-9 expression in non-small cell lung cancer cells

**DOI:** 10.18632/oncotarget.16837

**Published:** 2017-04-05

**Authors:** Jun An, Ying Xue, Meijun Long, Ge Zhang, Junhang Zhang, Hang Su

**Affiliations:** ^1^ Department of Cardiothoracic Surgery, The Third Affiliated Hospital of Sun Yat-sen University, Guangzhou, Guangdong 510630, China; ^2^ Department of Microbial and Biochemical Pharmacy, School of Pharmaceutical Sciences, Sun Yat-sen University, Guangzhou, Guangdong 510006, China; ^3^ Breast Cancer Center and Department of Thyroid and Breast Surgery, The Third Affiliated Hospital of Sun Yat-sen University, Guangzhou, Guangdong 510630, China; ^4^ Department of Radiation Oncology, The University of Texas Health Science Center at San Antonio, San Antonio, TX 78229, USA

**Keywords:** CCR2 antagonist, MMP-9, non-small cell lung cancer, viability, motility and invasion

## Abstract

Non-small cell lung cancer (NSCLC) is the most common type of lung cancer, which is the leading cancer killer in the world. Despite the recent advances in its diagnosis and therapy, the prognosis of NSCLC patients remains very poor, mainly due to the development of drug resistance and metastasis. Both the chemokine network and the matrix metalloproteinase (MMP) system play important roles in cancer cell metastasis. The disruption of CCL2/CCR2 chemokine signaling has been shown to suppress cancer cellviability and metastasis. CCL2-neutralizing antibodies, which have shown promising therapeutic efficacy in several cancer models, are not widely used due to technical issues. CCR2 antagonism has thus become an alternative method for cancer treatment. However, the effect of CCR2 antagonists on NSCLC progression remains poorly understood. Here, we investigated the effect of CCR2 antagonist (CAS445479-97-0) on the proliferation, migration and invasion of human lung adenocarcinoma A549 cells by using WST-1 cell viability assay, transwell migration assay, wound healing scratch assay and Matrigel invasion assay. We demonstrated that CCL2 treatment promoted A549 cell viability, motility and invasion by upregulating MMP-9 expression and that this induction was significantly suppressed by CAS 445479-97-0. Taken together, our data suggested that the CCR2 antagonist would be a potential drug for treating CCR2-positive NSCLC patients.

## INTRODUCTION

Non-small cell lung cancer (NSCLC) is the most common type of lung cancer, which is the leading cancer killer in the world [1, 2]. NSCLC patients can be generally classified into three stages: early (non-metastatic), locally advanced (confined to the thoracic cavity) and distant metastasis (outside of the thoracic cavity). Unfortunately, the prognosis of NSCLC patients remains very poor, despite the recent advances in therapy, probably due to the development of locally advanced or metastatic disease at the time of diagnosis [3]. Currently, the main therapeutic strategies for advanced and metastatic NSCLC are chemotherapy and specific mutagenic inhibitors for epidermal growth factor receptor (EGFR), anaplastic lymphoma kinase (ALK), c-met and Kras [4]. However, specific mutagenic inhibitors are only suitable for the rare mutagenic cases [5], and most cases of NSCLC rapidly develop acquired resistance to chemotherapy [6]. Therefore, there is an urgent need for new therapeutic approaches for NSCLC patients.

Chemokines are a superfamily of small, soluble and secreted proteins. Chemokines and their receptors play roles in numerous physiological and pathological processes, including cancer development. A growing body of evidence shows that they coordinate the survival and metastasis of cancer cells [7, 8]. To date, approximately 50 human chemokines have been identified, and Chemokine (C-C motif) Ligand 2 (CCL2), also known as Monocyte Chemotactic Protein-1 (MCP-1), is a member of the CC chemokine subfamily. CCL2 was purified and cloned in 1989 from human gliomas and myelomonocytic cells by two independent research groups and was found to participate in recruiting and activating monocytes during acute inflammation and angiogenesis [9–11]. CCL2 is produced by endothelial cells and fibroblasts [12] and by a variety of activating cells, such as lymphocytes and macrophages [9]. Recent studies have reported that CCL2 is overexpressed in a majority of solid cancer types, including breast cancer, prostate cancer, esophageal carcinoma, colon cancer, pancreatic cancer, ovarian cancer [8, 13–17] and NSCLC [18].

CC Chemokine Receptor 2 (CCR2) was mainly activated to the sites of inflammation by CCL2 in various cell types, including monocytes, macrophages, dendritic cells, and memory T cells [19–21]. Recently, we reported that CCR2 transduced tumor antigen-specific CD8^+^ T cell trafficking by CCL2 expression in lung cancer cells, which potentiated its *in vivo* anti-lung cancer reactivity [22]. CCR2 is expressed by a variety of tumor cell types [23]. The altered expression of CCL2 and CCR2 was found in NSCLC cells and was correlated with sex, smoking habits, histology and tumor size. In patients with NSCLC, positive CCL2 expression was observed more frequently in men than in women, in never-smokers than in smokers, in adenocarcinoma than in other histological types, and in smaller tumors among the patients with NSCLC. However, there was no relationship of tumor CCR2 expression with gender, smoking habits, histologic type of tumor and tumor size [18, 24]. However, its roles in NSCLC development remain unclear. Because CCL2 is a chemokine with a wide range of features, the blockade of CCL2 may have unwanted defects. For example, CCL2 blockade may target CCL2-dependent leukocyte adhesion and activate the endothelial and transendothelial migration of leukocytes at sites of inflammation [25]. Recent studies have indicated that CCR2, but not CCL2, regulates CCL2-induced breast cancer cell survival and motility through MAPK- and Smad3-dependent mechanisms [8].

In contrast, metastatic cancer cells that are distant from the primary tumor must first cross the basement membrane (BM), which is a network of extracellular matrix (ECM). Matrix metalloproteinases (MMPs) play an important role in cancer cell metastasis, as particularly observed for the roles MMP-2 and MMP-9 in the degradation of ECM [26, 27]. A recent study showed that crosstalk between the MMP system and the chemokine network plays a role in cancer cell metastasis. Both the chemokine system and MMPs are currently being evaluated as targets in anti-cancer therapy and may have potential therapeutic implications [28].

In this study, we examined the expression of CCL2 and its receptor CCR2 in various human NSCLC cell lines and investigated the effect of the CCL2/CCR2 interaction in A549 cell proliferation, migration and invasion *in vitro*. We demonstrated that CCL2 promoted A549 cell proliferation, migration and invasion by upregulating MMP-9 expression and that CCR2 antagonist treatment can inhibit CCL2-mediated A549 cell viability, motility and invasion by downregulating MMP-9 expression. Our data indicated that targeting CCR2 with an antagonist would be an attractive strategy to ameliorate cancer cell viability, motility and invasion in CCR2-positive NSCLC patients.

## RESULTS

### The expression of CCL2 and its receptor CCR2 in human NSCLC cell lines

**Figure 1:**
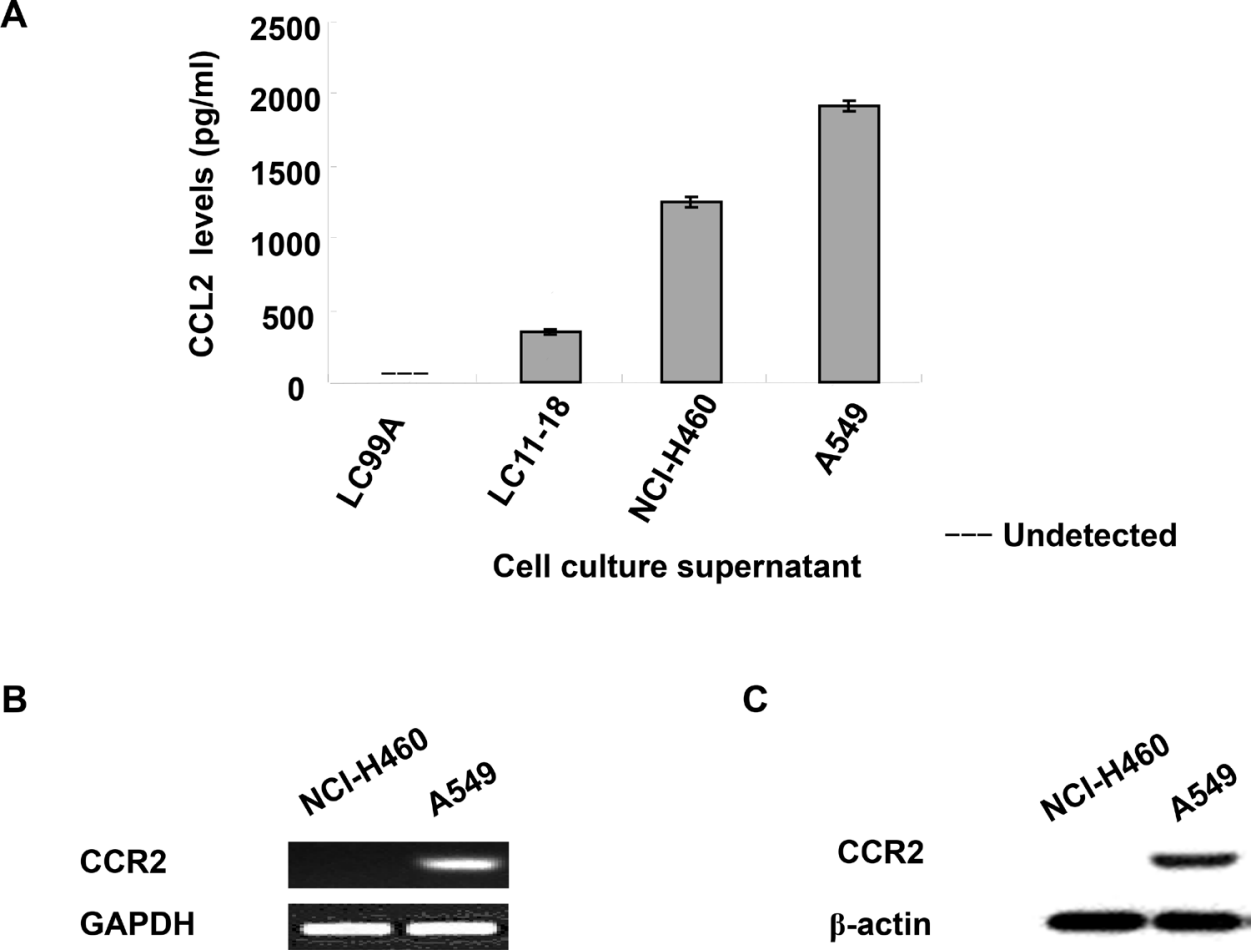
Expression of CCL2 and CCR2 in human NSCLC cell lines **(A)** Culture medium from LC99A, LC11-18, NCI-H460 and A549 cell lines was collected and CCL2 level was measured by ELISA; **(B)** mRNA expression of CCR2 in NCI-H460 and A549 cells were determined by RT-PCR; **(C)** the protein level of CCR2 in NCI-H460 and A549 cell were detected by Western blot.

We first examined the protein expression level of CCL2 in the culture media of different human NSCLC cell lines, including LC99A, LC11-18, NCI-H460 and A549. CCL2 expression was detected in the media of LC11-18, NCI-H460 and A549. As shown in Figure 1A, A549 secreted the highest amount of CCL2 among all of the examined NSCLC cell lines. We further compared the mRNA and protein levels of the CCL2 receptor, CCR2, between A549 and NCI-H460, both of which secreted high levels of CCL2 into the culture media. The RT-PCR (Figure 1B and Supplementary Figure 1) and Western blotting (Figure 1C) results suggested a much greater degree of CCR2 expression in A549 cells, whereas in NCI-H460, the level of CCR2 was undetectable.

### CCL2 promoted A549 cell proliferation, migration and invasion *in vitro*

**Figure 2:**
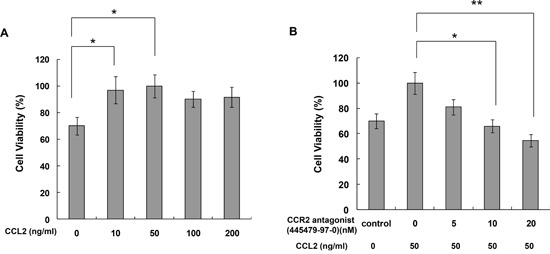
CCR2 antagonist inhibited CCL2-mediated A549 cell proliferation *in vitro* **(A)** A549 cells were treated with 0-200 ng/ml CCL2 for 72h and then subjected to cell viability assay; **(B)** A549 cells were treated with 50 ng/ml CCL2 for 72h with or without pretreatment of CCR2 antagonist (CAS 445479-97-0) (0-20 nM). Cell viability was then measured by WST-1 assay. Bars, SD (*, p<0.05, **, p<0.01).

**Figure 3:**
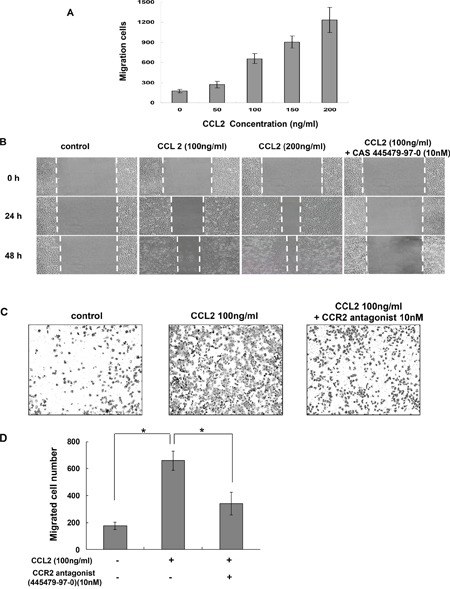
CCR2 antagonist inhibited CCL2-mediated A549 cell migration *in vitro* **(A)** Transwell migration assay. A549 cells were set in upper chamber of a 24-well transwell. RPMI 1640 with 10% FCS culture medium containing a serial dilution of CCL2 was added to the bottom well. After 4 h incubation, cells were fixed and the migration cell numbers for each group were counted. **(B)** Wound healing scratch assay. A549 cells were pretreated with or without CCR2 antagonist, CAS 445479-97-0 (10 nM) for 2 h. Then an artificial scratch wound was created using a p10 pipet tip. Cells were then incubated with serum free medium containing variable concentration of CCL2 and monitored at indicated time points. **(C)** Representative photographs of transwell migration assay with A549 cells in the presence of CCL2 (100 ng/ml) with or without pretreatment of CAS 445479-97-0. **(D)** The migration cell numbers for each group were calculated and are shown. The difference (*P < 0.05) was analyzed by Two-tailed paired Student's t-test.

**Figure 4:**
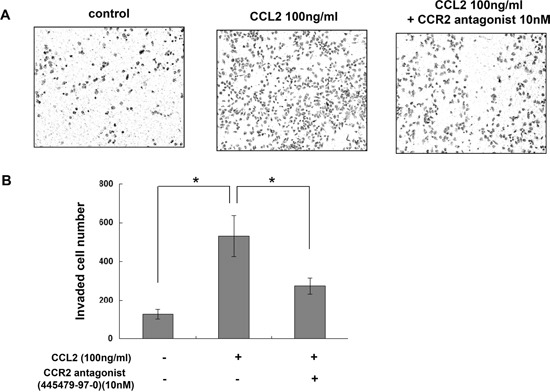
CCR2 antagonist inhibited CCL2-mediated A549 cell invasion *in vitro* **(A)** Representative photographs of invasion assay with A549 cells in the presence of CCL2 (100 ng/ml) with or without pretreatment of 10 nM of CAS 445479-97-0. **(B)** The invaded cell numbers for each group were calculated and are shown. The difference (*P < 0.05) was analyzed by Two-tailed paired Student's t-test.

To determine whether CCL2 is a regulator of A549 cell proliferation, cells were treated with different concentrations of rhCCL2 (0, 10, 50, 100 and 200 ng/ml) and examined for cell viability using WST-1 assay. We found that rhCCL2 was able to increase the cell proliferation and that this effect became saturated at 50 ng/ml (Figure 2A), partially due to the high background level of CCL2 secreted by A549 itself. To test whether CCL2 is a regulator of A549 cell migration, we tested the effect of a serial dilution of rhCCL2 on A549 cell migration using a cell monolayer transwell experiment [22] and wound healing scratch assay. The number of CCL2-mediated migrated A549 cells increased markedly in a dose-dependent manner (Figure 3A). In addition, CCL2 increased the wound healing migration activity of A549 cells in a dose-dependent manner (Figure 3B). We further utilized a Matrigel invasion assay to test whether CCL2 is a regulator of A549 cell invasion. As shown in Figure 4A and 4B, the number of CCL2-mediated invaded A549 cells increased significantly.

Moreover, to determine whether CCR2 is essential for the CCL2-mediated viability and motility of NSCLC cells. The NCI-H460 cells, which expressed undetectable CCR2 (Figure 1B and 1C and Supplementary Figure 1), were also examined. However, no significant changes was observed in proliferation and migration of NCI-H460 cells, regardless of the presence or absence of CCL2 (Supplementary Figures 2–3), which suggests that CCL2 mediates its major effects through its receptor CCR2 in NCI-H460 cells.

The disruption of CCL2/CCR2 chemokine signaling has been shown to suppress cancer cell proliferation, migration and invasion. Since CCL2 is a chemokine with a wide range of features, the blockade of CCL2 may have unwanted defects. Therefore, further experiments were performed to verify whether CCR2 antagonism inhibited CCL2-mediated A549 cell proliferation, migration and invasion *in vitro*.

### CCR2 antagonist inhibited CCL2-mediated A549 cell proliferation, migration and invasion *in vitro*

As shown in Figure 2B, pretreatment with CCR2 antagonist (CAS 445479-97-0) reversed the induction of A549 cell proliferation by rhCCL2 in a dose-dependent manner. As shown in Figure 3C and 3D, CAS 445479-97-0 largely abrogated CCL2-mediated migration in A549 cells. In addition, CAS 445479-97-0 reduced the CCL2-induced wound healing activity in A549 cells (Figure 3B). As shown in Figure 4A and 4B, whereas rhCCL2 increased cell invasion by 4.2-fold, pretreatment with CAS 445479-97-0 decreased rhCCL2-mediated A549 cells invasion to 51.6±16.0%. Together, our results suggested that CCR2 antagonist inhibited CCL2-mediated A549 cell proliferation, migration and invasion *in vitro*.

### CCL2 promoted A549 cell migration and invasion via the upregulation of MMP-9 expression

**Figure 5:**
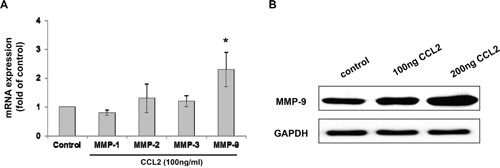
CCL2 increased the expression of MMP-9 in A549 cell **(A)** A549 cells were treated with CCL2 (100 ng/ml) for 24 h, the mRNA levels of MMP-1, -2, -3 and -9 were detected by qRT-PCR. **(B)** A549 cells were treated with CCL2 (0-200 ng/ml) for 24 h, the protein level of MMP-9 was examined by Western blot.

**Figure 6:**
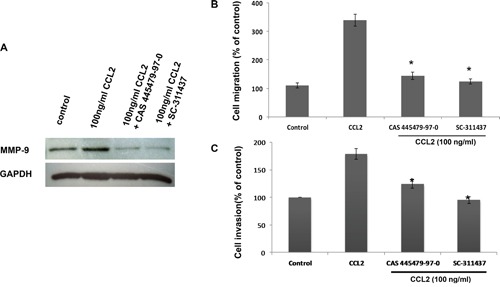
CCR2 antagonist inhibited CCL2-mediated A549 cell migration and invasion via downregulating MMP-9 expression A549 cells were pretreating with 10 nM CCR2 antagonist (CAS 445479-97-0) for 24 h or 5uM MMP-9 inhibitor (sc-311437) for 30 min, followed by stimulation with CCL2 for 24 h. The protein level of MMP-9 was then detected by Western blot **(A)**, the migration and invasion activity of A549 cells were assessed by transwell **(B)** and Matrigel assays **(C)**, respectively. *p < 0.05 represents statistically significant differences between the group pretreated with CCR2 antagonist or MMP-9 inhibitor and the CCL2-treated group.

Previous studies have shown that MMPs play an important role in cancer cell metastasis, with significant expression of MMP-2 and MMP-9 in A549 cells [29]. Therefore, we examined whether the MMPs involved in CCL2-mediated A549 cell migration and invasion. After treatment with CCL2 (100 ng/ml) for 24 h, the mRNA expression of MMP-1, -2, -3 and -9 was examined by qRT-PCR. The results showed that the mRNA expression of MMP-2, -3 and -9 was increased but that only the mRNA expression of MMP-9 was significantly increased (Figure 5A). Further increases in the protein levels of MMP-9 in A549 cells after 24 h of treatment with CCL2 (100 ng/ml) were examined by Western blot (Figure 5B). Since our previous experiments demonstrated that CCL2 treatment promoted A549 cell migration and invasion activity and that the protein level of CCL2-mediated MMP-9 increased in a dose-dependent manner (Figure 5B), further experiments were performed to verify whether CCL2-mediated A549 cell migration and invasion were regulated by MMP-9. As shown in Figure 6B and 6C, after the downregulation of MMP-9 expression with MMP-9 inhibitor I (sc-311437, 5 μM) for 30 min, the number of CCL2-mediated that migrated into and invaded A549 cells decreased significantly. Taken together, our results suggested that the upregulation of MMP-9 expression played an important role in CCL2-mediated A549 cell migration and invasion *in vitro*.

### CCR2 antagonist inhibited CCL2-mediated A549 cells migration and invasion by downregulating MMP-9 expression

As shown in Figure 6A, the protein level of CCL2-induced MMP-9 was reduced by pretreatment with CCR2 antagonist (CAS 445479-97-0, 10 nM, 24 h) or MMP-9 inhibitor I (sc-311437, 5 μM, 30 min). As expected, pretreatment with CAS 445479-97-0 (10 nM, 24 h) inhibited CCL2-mediated A549 cell migration by 58% and invasion by 30% (Figure 6B and 6C). Taken together, our results suggested that CCR2 antagonist inhibited CCL2-mediated A549 cell migration and invasion by downregulating MMP-9 expression through the CCR2 receptor *in vitro*.

## DISCUSSION

Increasing evidence points to the vital effects of CCL2/CCR2 on the proliferative and metastatic properties of cancer cells (Supplementary Figure 4) [7, 8, 11]. The current study investigated the anti-proliferative, anti-motile and anti-invasive activities of an antagonist against CCR2, CAS 445479-97-0, by blocking the CCL2/CCR2 axis interaction and downregulating MMP-9 protein expression in human lung adenocarcinoma A549 cells *in vitro*. Here, we found the following: first, CCL2/CCR2 interaction promoted A549 cell proliferation, migration and invasion, although to a lesser extent compared with those in other cancer cells, such as prostate and breast cancer cells [30, 31], which was indicative of tissue-specific functions for CCL2 signaling. Second, *in vitro* CCL2-mediated A549 cell proliferation, migration and invasion by upregulating MMP-9 expression could be suppressed by CCR2 antagonist. Third, the upregulation of MMP-9 protein expression played an important role in CCL2-induced A549 cell motility and invasion *in vitro*.

The disruption of CCL2/CCR2 chemokine signaling has been shown to suppress cancer cell viability and metastasis. By targeting CCL2 directly, neutralizing antibodies rather than antagonists are usually used. CCL2 neutralizing antibodies, which have shown promising therapeutic efficacy in several cancer models [16, 32]. However, the production and storage for antibodies will be challenging and costly than chemical antagonists. Besides the limitation on the cost, CCL2 antibodies have shown some limitation for cancer treatment, as the removal of anti-CCL2 antibody in cancer treatment was found leading to an increased metastatic burden in an orthotropic mammary tumor model [33], While the cessation of CCR2 antagonist (RDC018, GlaxoSmithKline) did not enhance malignant progression in hepatocellular carcinoma postsurgical recurrence mice models. Moreover, animals receiving CCR2 antagonist have a much longer survival rate [34].

Based on this knowledge, we focused on CCR2 and investigated the effects of an antagonist CAS 445479-97-0 against CCR2 on the proliferation, migration and invasion of A549 cells *in vitro*. CCR2 antagonist, CAS 445479-97-0, is a cell-permeable cis-diamidocyclohexyl urea compound with high affinity binding to Thr292, which is adjacent to the key receptor residue Glu291. Herein, we examined the potential use of the CCR2-specific small molecule inhibitor (CAS 445479-97-0) in cancer therapy. Our results suggested that the blockade of the CCR2 pathway inhibited the viability, motility and invasion of CCR2-positive human A549 lung cancer cells *in vitro*. Furthermore, we found that the *in vitro* use of CCR2 antagonist or MMP-9 inhibitor could suppress the CCL2-induced upregulation of MMP-9 expression and reduce the migration and invasion of A549 cells *in vitro*.

It is well known that MMPs play a crucial role in the invasion, migration and angiogenesis of cancer cells and that MMP-9 is a major factor in cancer cell migration and invasion [35]. Our data indicated that treating A549 cells with CCR2 antagonist downregulated CCL2-enhanced MMP-9 protein expression and significantly reduced CCL2-induced cell migration and invasion *in vitro*. Therefore, MMP-9, as one of the mediators of the CCL2/CCR2 axis interaction, may cause the inhibition of cancer cell migration and invasion. The ERK1/ERK2 signaling pathway has been indicated in CCL2/CCR2-induced metastasis MMP-9 expression. Firstly, the association between CCL2/CCR2 axis and the MAPK pathways has been found in cervical cancer, breast cancer, colon carcinoma and melanoma cells [8, 14, 36]. Secondly, the expression of MMP-2 and MMP-9 can also be regulated by ERK1/2 in cancer cells [37, 38]. Lastly, the direct link of MMP-9 regulation to CCL2/CCR2 axis was proved to involve the activation of Ras, Raf-1, MEK, ERK, and NF-κB pathway [39]. A recent study also found that CCL2-CCR2 axis promotes nasopharyngeal carcinoma metastasis by activating ERK1/2-MMP2/9 pathway [40]. However, the detailed signaling transduction pathways through which CCL2/CCR2 regulates MMP-9 expression in A549 cells remain unknown. Interestingly, the blockade of the MMP-9 inhibitor I (sc-311437) reduced CCL2-mediated MMP-9 expression via an unidentified mechanism. In line with our results, a recent study by Shi et al. found that PinX1 inhibited NF-kB mediated MMP-9 expression, and that MMP-9 inhibitor I, sc-311437, brought down MMP-9 expression when PinX1 expression was suppressed [41]. It is likely that sc-311437 affect MMP-9 expression via an unknown mechanism, which involved NF-κB activity. Our data demonstrated that CCR2 blockade by its antagonist downregulated MMP-9 protein expression. In addition, blockade via the MMP-9 inhibitor reduced CCL2-mediated cell migration and invasion. Therefore, MMP-9 may be one of the mediators of the CCL2/CCR2 axis. Here, we proposed a new possible mechanism for CCL2-induced A549 cell migration and invasion *in vitro* via the upregulation of MMP-9 expression.

Moreover, CCR2 was expressed in multiple immunosuppressive cells, such as CD4+FOXP3+ CCR2+ Treg cells [49], CCR2-expressing myeloid-derived suppressor cells (MDSCs) and tumor-associated macrophages (TAMs), which were recruited to the tumor site through the expression of CCR2 and play a critical role in tumor escape [50, 51]. Therefore, the blockade of CCR2 may decrease the recruitment of immunosuppressive cells to the tumor microenvironment and thereby prevent tumor progression.

As stated above, the tumorigenic features of CCL2/CCR2 signaling were described, but CCL2/CCR2 signaling also plays a protective role in antitumor effects, such as through the recruitment of type 1 cytotoxic gammadelta T lymphocytes to the tumor microenvironment [36]. Therefore, the CCL2/CCR2 reaction may have dual context-dependent effects in tumorigenesis: promoting the metastasis of tumors and providing anti-tumor effects at the same time. Further clarification is required to understand the targeting of the CCL2/CCR2 pathway in cancer therapy.

Previous studies have found that targeting the chemokine receptor could inhibit cancer cell proliferation, migration and invasion [32, 42, 43]. Our study also demonstrated that CCR2 antagonist suppressed the CCL2-mediated viability, motility and invasion of the NSCLC cell line A549 *in vitro* by downregulating MMP-9 expression. Therefore, the CCR2 antagonist may be a potential novel therapeutic option in the treatment of CCR2-positive NSCLC.

## MATERIALS AND METHODS

### Reagents

Mouse anti-human-CCR2 (clone: REA264), PE conjugated mcAb was purchased from Miltenyi Biotec (Auburn, CA, USA), Rabbit anti-MMP-9 pcAb was purchased from Bioworld Tech (St. Louis Park, MN, USA); Recombinant human CCL2 (rhCCL2) was purchased from Pepro Tech (Rocky Hill, NJ, USA), CCR2 antagonist (CAS 445479-97-0, sc-202525) and MMP-9 Inhibitor I (sc-311437) were purchased from Santa Cruz Biotechnology (Santa Cruz, CA, USA). All other reagents were purchased from Sigma-Aldrich (St. Louis, MO, USA) unless otherwise indicated in the text.

### Cell lines and cell culture

Human NSCLC cell lines used in this study including A549 (adenocarcinoma), NCI-H460 (large cell carcinoma), LC99A (large cell carcinoma origin) and LC11-18 (adenocarcinoma) [22] were cultured in RPMI 1640 medium supplement with 10% FCS, 0.1 mg/ml streptomycin and 100 U/ml penicillin G. The culture medium was replaced every 2 days. All cells were incubated at 37°C, 5% CO_2_ in a humidified incubator.

### Reverse transcription-polymerase chain reaction (RT-PCR)

Total RNA was extracted from each sample with an RNeasy Mini Kit (QIAGEN, Hilden, Germany) and reverse transcribed using the SuperScript II reverse transcriptase (Invitrogen, Calsbard, CA, USA) in accordance with the manufacturer's instructions. The PCR primers for CCR2 mRNA amplification were 5^'^-CTGTGTTTGCTTCTGTCC-3^'^ (forward) and 5^'^-CCCTATGCCTCTTCTTCTC-3^'^ (reverse); for CCL2, 5^'^-CTTCTGTGCCTGCTGCTCATAG-3^'^ (forward), and 5′-GTTTGGGTTTGCTTGTCCAG-3′ (reverse). The mRNA expression of Glyceraldehyde-3-phosphatedehydrogenase (GAPDH) was served as an internal control. PCR conditions were 2 min at 95°C followed by 25 cycles of 95°C for 1 min, 60°C for 30 s and 72°C for 1 min. The PCR product of each sample was analyzed by electrophoresis in 1.5% agarose gel andvisualized by ethidium bromide staining.

### Quantitative real-time PCR (qRT-PCR)

The MMP-9 mRNA expression level in A549 cells after treatment with CCL2 for 24h were detected by qRT-PCR as described previously [22]. Briefly, total RNA was extracted from each sample with an RNeasy Mini Kit (Qiagen, Hilden, Germany) and cDNA was synthesized. qRT-PCR for MMP-9 mRNA was performed using a QuantiTect SYBER Green PCR kit (Qiagen, Hilden, Germany) in accordance with the manufacturer's instructions. The qRT-PCR primers for MMP-9 mRNA amplification were 5′-GCCCTGGAACTCACACGACA-3′ (forward), and 5′-TTGGAAACTCACACGCCAGAAG-3′ (reverse). GAPDH was served as an internal control. The cycling conditions were 10 min at 95°C followed by 40 cycles of 95°C for 15 s and 60°C for 60 s. The threshold was set above the non-template control background and within the linear phase of target gene amplification to calculate the cycle number at which the transcript was detected.

### Western blot

CCR2 expression was examined by Western blotting as described elsewhere [44]. Briefly, cell lysates from 1×10^6^ cells were collected. After electrophoresis in 12.5% SDS–polyacrylamide gel, blots were reacted with anti-human CCR2 mcAb. For analysis of protein level of MMP-9, cell lysates from 1 × 10^6^ A549 cells treated with CCL2 or pretreated with CCR2 antagonist or MMP-9 inhibitor I, the blots were reacted with anti-MMP-9 pcAb. After incubation with the horseradish peroxidase-conjugated secondary antibody, membranes were extensively washed, and the blots were visualized using an enhanced system (ECL kit, Santa Cruz, CA, USA). Anti-β-actin mcAb (Cambridge, MA, USA) was used as loading control.

### Proliferation assay

Cells were seeded into 96-well plates at a density of 3,000 cells per well in RPMI 1640 with 10% FCS culture medium and maintained at 37°C and 5% CO_2_ humidified incubator for 48h. The cells were then starved by replacing the medium with RPMI 1640 containing 1% FCS. Twelve hours after starvation, the medium was removed and replaced with RPMI 1640 containing rhCCL2at (0, 10, 50, 100 and 200 ng/ml). For blocking experiment, CCR2 antagonist (CAS 445479-97-0) (0 to 20 nM) was added to the cells 30 min before the addition of rhCCL2 (50 ng/ml). WST-1 Cell viability assay (BD Clontech, Mountain View, CA, USA) were performed 72 h after treatment. Briefly, 10 μl/well WST-1 was added to each well and incubated for 4 h. The absorbance was measured at 450 nm using an automated microplate reader Elx808 (Bio-Tek Instruments, Winooski, VT).

### Migration assay

The migration assay was conducted via transwell experiments. In brief, 2×10^5^ A549 cells were placed in the upper well of a 3-mm pore-size 24-well transwell plate (Corning Incorporation, Corning, NY, USA). 500 ul of RPMI 1640 with 10% FCS culture medium containing a serial dilution of CCL2 was added to the bottom well. After four hours of incubation, the cells in the bottom well were fixed in 4% paraformaldehyde for 30 min, stained using hematoxylin andcounted under a microscope. A blocking experiment was conducted using a CCR2 antagonist, CAS 445479-97-0 (10 nM), which was added to the cells before the cells were placed in the upper compartment.

### Wound healing scratch assay

The wound healing scratch assay was used to evaluate the migration of A549 cells with or without CCL2 and anti-CCR-2 *in vitro*. A549 cells were seeded in six-well plates at a density of 1×10^6^ per well with complete medium. When cell confluency reaches 100%, the monolayers were starved overnight in RPMI1640 without newborn bovine serums, then an artificial scratch wound was created using a p10 pipet tip. The wells were washed with PBS to remove cell debris and replaced with fresh RPMI1640 containing variable concentration of CCL2 and monitored. For blocking assays, the scratch wound was created after 2 h of the CCR2 antagonist (CAS 445479-97-0) (20 μg/ml) pretreatment. Cell migration was photographed and the width of the wound was measured.

### Invasion assay

Ability of cell invasion was evaluated by the Cultrex 24-well Transwell BME cell invasion assay (Trevigen Inc., Gaithersburg, MD, USA). Briefly, a 24-well unit with 8-mm polycarbonate nucleopore filters (Corning) was evenly coated with 100ul of basement membrane extract coating solution at 37°C for 4 h. 2×10^5^ A549 cells in serum free medium were placed in the upper compartment. RPMI 1640 medium supplied with 0.5% FBS with 100 ng/ml of rhCCL2 was added to the lower compartment. After 24 hours incubation, cells that had not invaded were removed with a cotton swab. Cells that had invaded to the lower surface of the membrane were fixed with 4% formaldehyde and stained with crystal violet and observed under a microscope. For the blocking experiment, 10 nM CCR2 antagonist (CAS 445479-97-0) was added to the cells before the cells were loaded in the upper compartment. Five microscopic fields were randomly selected to calculate the count of the invaded cells. The relative number of cell that penetrated the basement membrane was used to denote the invasion ability of the cells.

### ELISA

CCL2 in the culture supernatant was measured using an ELISA kit (R&D Systems, Minneapolis, MN, USA). 1×10^5^ cells were incubated in 24 well plates for 24 hours. Conditioned media generated from the indicated cell lines were analyzed according to the manufacturer's protocol. The reaction was stopped with 1M HCl, and absorbance was read at 450 nm using a BioTek microplate reader (EL-309, Vermont, USA).

### Statistical analysis

All the experiments were carried out in triplicate. Two-tailed paired Student's t-test was performed to find the significance between two groups. Values of *p*< 0.05 were considered to represent statistically significant differences.

## SUPPLEMENTARY FIGURES



## References

[1] Siegel R, Naishadham D, Jemal A (2013). Cancer statistics, 2013. CA Cancer J Clin.

[2] Goldstraw P, Ball D, Jett JR, Le Chevalier T, Lim E, Nicholson AG, Shepherd FA (2011). Non-small-cell lung cancer. Lancet.

[3] Yang P (2009). Epidemiology of lung cancer prognosis: quantity and quality of life. Methods Mol Biol.

[4] Sasaki T, Koivunen J, Ogino A, Yanagita M, Nikiforow S, Zheng W, Lathan C, Marcoux JP, Du J, Okuda K, Capelletti M, Shimamura T, Ercan D (2011). A novel ALK secondary mutation and EGFR signaling cause resistance to ALK kinase inhibitors. Cancer Res.

[5] Lynch TJ, Bell DW, Sordella R, Gurubhagavatula S, Okimoto RA, Brannigan BW, Harris PL, Haserlat SM, Supko JG, Haluska FG, Louis DN, Christiani DC, Settleman J (2004). Activating mutations in the epidermal growth factor receptor underlying responsiveness of non-small-cell lung cancer to gefitinib. N Engl J Med.

[6] Rosell R, Felip E (2001). Predicting response to paclitaxel/carboplatin-based therapy in non-small cell lung cancer. Semin Oncol.

[7] Conti I, Rollins BJ (2004). CCL2 (monocyte chemoattractant protein-1) and cancer. Semin Cancer Biol.

[8] Fang WB, Jokar I, Zou A, Lambert D, Dendukuri P, Cheng N (2012). CCL2/CCR2 chemokine signaling coordinates survival and motility of breast cancer cells through Smad3 protein- and p42/44 mitogen-activated protein kinase (MAPK)-dependent mechanisms. J Biol Chem.

[9] Zachariae CO, Anderson AO, Thompson HL, Appella E, Mantovani A, Oppenheim JJ, Matsushima K (1990). Properties of monocyte chemotactic and activating factor (MCAF) purified from a human fibrosarcoma cell line. J Exp Med.

[10] Tangirala RK, Murao K, Quehenberger O (1997). Regulation of expression of the human monocyte chemotactic protein-1 receptor (hCCR2) by cytokines. J Biol Chem.

[11] Salcedo R, Ponce ML, Young HA, Wasserman K, Ward JM, Kleinman HK, Oppenheim JJ, Murphy WJ (2000). Human endothelial cells express CCR2 and respond to MCP-1: direct role of MCP-1 in angiogenesis and tumor progression. Blood.

[12] Strieter RM, Wiggins R, Phan SH, Wharram BL, Showell HJ, Remick DG, Chensue SW, Kunkel SL (1989). Monocyte chemotactic protein gene expression by cytokine-treated human fibroblasts and endothelial cells. Biochem Biophys Res Commun.

[13] Scotton C, Milliken D, Wilson J, Raju S, Balkwill F (2001). Analysis of CC chemokine and chemokine receptor expression in solid ovarian tumours. Br J Cancer.

[14] Wolf MJ, Hoos A, Bauer J, Boettcher S, Knust M, Weber A, Simonavicius N, Schneider C, Lang M, Sturzl M, Croner RS, Konrad A, Manz MG (2012). Endothelial CCR2 signaling induced by colon carcinoma cells enables extravasation via the JAK2-Stat5 and p38MAPK pathway. Cancer Cell.

[15] Zhang J, Lu Y, Pienta KJ (2010). Multiple roles of chemokine (C-C motif) ligand 2 in promoting prostate cancer growth. J Natl Cancer Inst.

[16] Qian BZ, Li J, Zhang H, Kitamura T, Zhang J, Campion LR, Kaiser EA, Snyder LA, Pollard JW (2011). CCL2 recruits inflammatory monocytes to facilitate breast-tumour metastasis. Nature.

[17] Monti P, Leone BE, Marchesi F, Balzano G, Zerbi A, Scaltrini F, Pasquali C, Calori G, Pessi F, Sperti C, Di Carlo V, Allavena P, Piemonti L (2003). The CC chemokine MCP-1/CCL2 in pancreatic cancer progression: regulation of expression and potential mechanisms of antimalignant activity. Cancer Res.

[18] Zhang XW, Qin X, Qin CY, Yin YL, Chen Y, Zhu HL (2013). Expression of monocyte chemoattractant protein-1 and CC chemokine receptor 2 in non-small cell lung cancer and its significance. Cancer Immunol Immunother.

[19] Charo IF (1999). CCR2: from cloning to the creation of knockout mice. Chem Immunol.

[20] Mack M, Cihak J, Simonis C, Luckow B, Proudfoot AE, Plachy J, Bruhl H, Frink M, Anders HJ, Vielhauer V, Pfirstinger J, Stangassinger M, Schlondorff D (2001). Expression and characterization of the chemokine receptors CCR2 and CCR5 in mice. J Immunol.

[21] Kuroda T, Kitadai Y, Tanaka S, Yang X, Mukaida N, Yoshihara M, Chayama K (2005). Monocyte chemoattractant protein-1 transfection induces angiogenesis and tumorigenesis of gastric carcinoma in nude mice via macrophage recruitment. Clin Cancer Res.

[22] Asai H, Fujiwara H, An J, Ochi T, Miyazaki Y, Nagai K, Okamoto S, Mineno J, Kuzushima K, Shiku H, Inoue H, Yasukawa M (2013). Co-introduced functional CCR2 potentiates in vivo anti-lung cancer functionality mediated by T cells double gene-modified to express WT1-specific T-cell receptor. PLoS One.

[23] Wang B, Chen G, Zhou J, Wu P, Luo D, Huang X, Zhu T, Han Z, Xu G, Wang S, Lu Y, Ma D (2007). Deletion of the intracellular domain of coxsackie and adenovirus receptor (CAR) enhances the expression of itself and boosts the efficiency of current adenovirus-mediated gene therapy in ovarian cancer cell lines in vitro. Cancer Lett.

[24] Li X, Tai HH (2013). Activation of thromboxane A2 receptor (TP) increases the expression of monocyte chemoattractant protein -1 (MCP-1)/chemokine (C-C motif) ligand 2 (CCL2) and recruits macrophages to promote invasion of lung cancer cells. PLoS One.

[25] Luster AD, Alon R, von Andrian UH (2005). Immune cell migration in inflammation: present and future therapeutic targets. Nat Immunol.

[26] Egeblad M, Werb Z (2002). New functions for the matrix metalloproteinases in cancer progression. Nat Rev Cancer.

[27] Loffek S, Schilling O, Franzke CW (2011). Series “matrix metalloproteinases in lung health and disease”: biological role of matrix metalloproteinases: a critical balance. Eur Respir J.

[28] Hatfield KJ, Reikvam H, Bruserud O (2010). The crosstalk between the matrix metalloprotease system and the chemokine network in acute myeloid leukemia. Curr Med Chem.

[29] Lin SS, Lai KC, Hsu SC, Yang JS, Kuo CL, Lin JP, Ma YS, Wu CC, Chung JG (2009). Curcumin inhibits the migration and invasion of human A549 lung cancer cells through the inhibition of matrix metalloproteinase-2 and -9 and Vascular Endothelial Growth Factor (VEGF). Cancer Lett.

[30] Zhang J, Patel L, Pienta KJ (2010). CC chemokine ligand 2 (CCL2) promotes prostate cancer tumorigenesis and metastasis. Cytokine Growth Factor Rev.

[31] Ueno T, Toi M, Saji H, Muta M, Bando H, Kuroi K, Koike M, Inadera H, Matsushima K (2000). Significance of macrophage chemoattractant protein-1 in macrophage recruitment, angiogenesis, and survival in human breast cancer. Clin Cancer Res.

[32] Lu X, Kang Y (2009). Chemokine (C-C motif) ligand 2 engages CCR2+ stromal cells of monocytic origin to promote breast cancer metastasis to lung and bone. J Biol Chem.

[33] Bonapace L, Coissieux MM, Wyckoff J, Mertz KD, Varga Z, Junt T, Bentires-Alj M (2014). Cessation of CCL2 inhibition accelerates breast cancer metastasis by promoting angiogenesis. Nature.

[34] Li X, Yao W, Yuan Y, Chen P, Li B, Li J, Chu R, Song H, Xie D, Jiang X, Wang H (2017). Targeting of tumour-infiltrating macrophages via CCL2/CCR2 signalling as a therapeutic strategy against hepatocellular carcinoma. Gut.

[35] Dagouassat M, Suffee N, Hlawaty H, Haddad O, Charni F, Laguillier C, Vassy R, Martin L, Schischmanoff PO, Gattegno L, Oudar O, Sutton A, Charnaux N (2010). Monocyte chemoattractant protein-1 (MCP-1)/CCL2 secreted by hepatic myofibroblasts promotes migration and invasion of human hepatoma cells. Int J Cancer.

[36] Gatti L, Sevko A, De Cesare M, Arrighetti N, Manenti G, Ciusani E, Verderio P, Ciniselli CM, Cominetti D, Carenini N, Corna E, Zaffaroni N, Rodolfo M (2014). Histone deacetylase inhibitor-temozolomide co-treatment inhibits melanoma growth through suppression of Chemokine (C-C motif) ligand 2-driven signals. Oncotarget.

[37] McCawley LJ, Li S, Wattenberg EV, Hudson LG (1999). Sustained activation of the mitogen-activated protein kinase pathway. A mechanism underlying receptor tyrosine kinase specificity for matrix metalloproteinase-9 induction and cell migration. J Biol Chem.

[38] Kumar B, Koul S, Petersen J, Khandrika L, Hwa JS, Meacham RB, Wilson S, Koul HK (2010). p38 mitogen-activated protein kinase-driven MAPKAPK2 regulates invasion of bladder cancer by modulation of MMP-2 and MMP-9 activity. Cancer Res.

[39] Tang CH, Tsai CC (2012). CCL2 increases MMP-9 expression and cell motility in human chondrosarcoma cells via the Ras/Raf/MEK/ERK/NF-kappaB signaling pathway. Biochem Pharmacol.

[40] Yang J, Lv X, Chen J, Xie C, Xia W, Jiang C, Zeng T, Ye Y, Ke L, Yu Y, Liang H, Guan XY, Guo X (2016). CCL2-CCR2 axis promotes metastasis of nasopharyngeal carcinoma by activating ERK1/2-MMP2/9 pathway. Oncotarget.

[41] Shi M, Cao M, Song J, Liu Q, Li H, Meng F, Pan Z, Bai J, Zheng J (2015). PinX1 inhibits the invasion and metastasis of human breast cancer via suppressing NF-kappaB/MMP-9 signaling pathway. Mol Cancer.

[42] Liang Z, Wu H, Reddy S, Zhu A, Wang S, Blevins D, Yoon Y, Zhang Y, Shim H (2007). Blockade of invasion and metastasis of breast cancer cells via targeting CXCR4 with an artificial microRNA. Biochem Biophys Res Commun.

[43] Loberg RD, Ying C, Craig M, Day LL, Sargent E, Neeley C, Wojno K, Snyder LA, Yan L, Pienta KJ (2007). Targeting CCL2 with systemic delivery of neutralizing antibodies induces prostate cancer tumor regression in vivo. Cancer Res.

[44] An J, Fujiwara H, Suemori K, Niiya T, Azuma T, Tanimoto K, Ochi T, Akatsuka Y, Mineno J, Ozawa H, Ishikawa F, Kuzushima K, Yasukawa M (2011). Activation of T-cell receptor signaling in peripheral T-cell lymphoma cells plays an important role in the development of lymphoma-associated hemophagocytosis. Int J Hematol.

