# Supplementary material for: Targeting CCR2 with its antagonist suppresses viability, motility and invasion by downregulating MMP-9 expression in non-small cell lung cancer cells
Source: Oncotarget. 2017 Apr 5;8(24):39230–40. doi: 10.18632/oncotarget.16837 (PMC5503609; doi:10.18632/oncotarget.16837)
Supplement: Supplementary file 1 [file oncotarget-08-39230-s001.pdf]

Targeting CCR2 with its antagonist suppresses viability, motility and invasion by downregulating MMP-9 expression in non-small cell lung cancer cells

SUPPLEMENTARY MATERIALS

SUPPLEMENTARY FIGURES

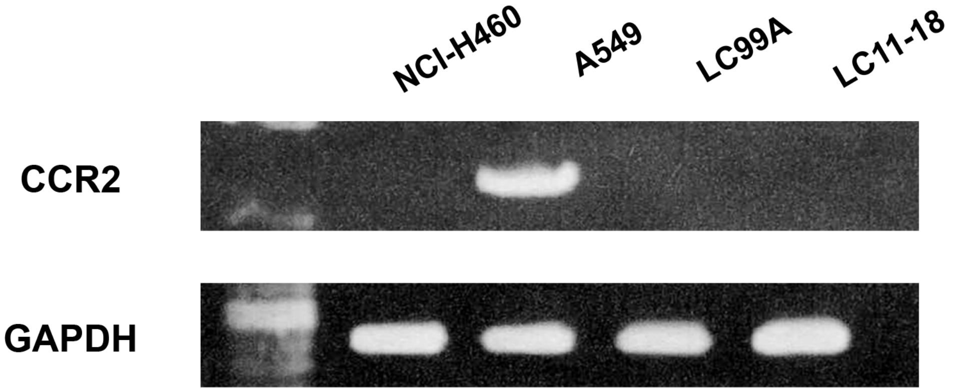

Supplementary Figure 1: Expression of CCR2 in human NSCLC cell lines. RT-PCR assays were performed to detect the mRNA expression of CCR2 in NCI-H460, A549, LC99A and LC11-18 cell.

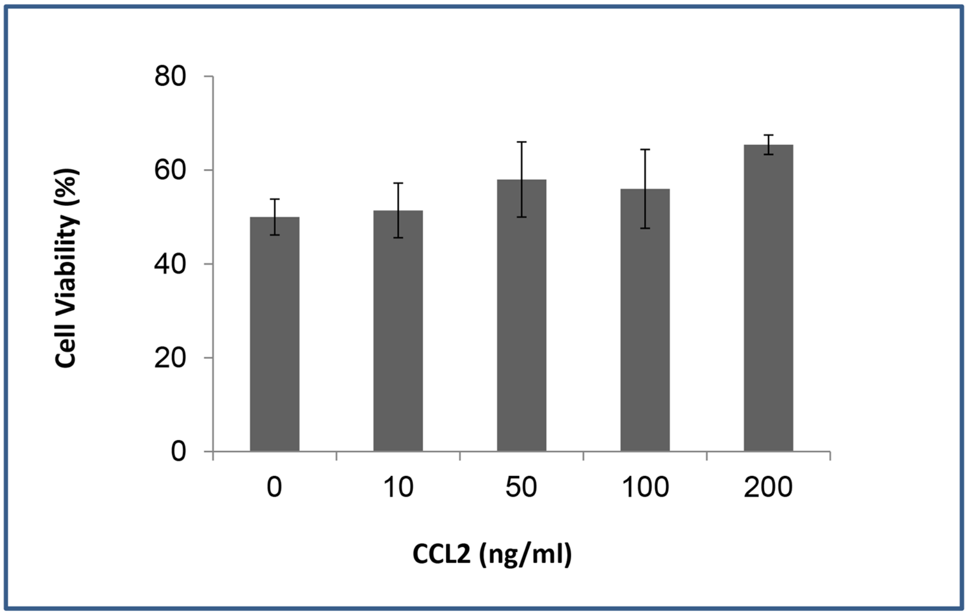

Supplementary Figure 2: CCL2-mediated NCI-H460 cell proliferation *in vitro*. Cell viability of NCI-H460 cells treated with 0-200 ng/ml CCL2. Cell viability was measured by WST-1 assay. Y-axis: mean percentage of medium control. Bars, SD. There is no significant difference between each group.

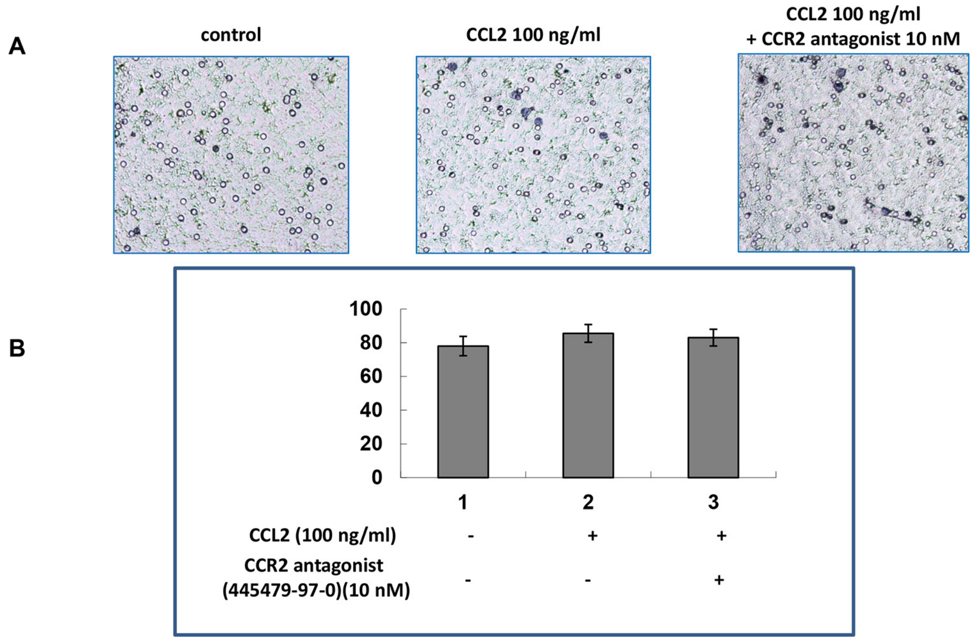

Supplementary Figure 3: CCL2-mediated NCI-H460 cell migration *in vitro*. (A) Representative photographs of migrated NCI-H460 cells treated with CCL2 (100 ng/ml) with or without pretreatment of CCR2 antagonist (CAS 445479-97-0) (10 nM) into the lower chamber. **(B)** Transwell assays were performed to detect the migration activity of NCI-H460 cell. The migrated number of the NCI-H460 cells did not change among the control, CCL2 with or without pretreatment of CCR2 antagonist group significantly.

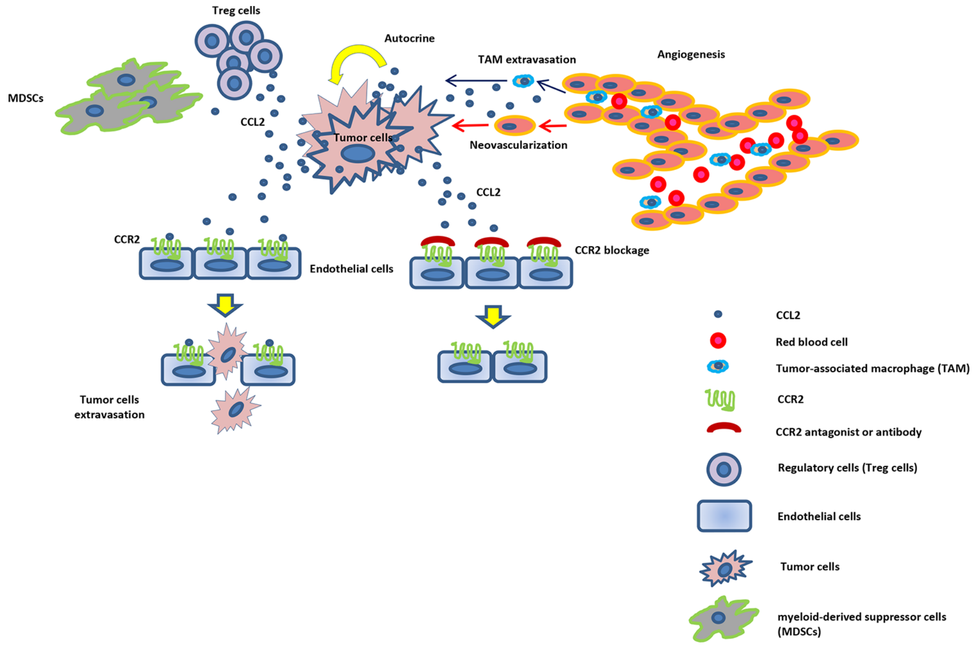

Supplementary Figure 4: CCL2/CCR2 axis in tumor microenvironment. CCL2 has been identified to affect the tumor cells by stimulating proliferation, survival and migration directly. CCL2/CCR2 chemokine axis also contributes to the development and progression of tumors via stimulating angiogenesis, tumor cells intravasation, MDSCs and TAMs recruitment by extravasation.
